# Pseudohypoaldosteronism Type II or Gordon Syndrome: A Rare Syndrome of Hyperkalemia and Hypertension With Normal Renal Function

**DOI:** 10.7759/cureus.52594

**Published:** 2024-01-19

**Authors:** FNU Manas, Sneha Singh

**Affiliations:** 1 Endocrinology, Henry Ford Health System, Detroit, USA; 2 Internal Medicine, Sunrise Hospital and Medical Center, Las Vegas, USA

**Keywords:** normal renal function, metabolic acidosis, thiazide diuretics, thiazide-sensitive na-cl cotransporter, hyperkalemia, hypertension, gordon syndrome, pseudohypoaldosteronism

## Abstract

Pseudohypoaldosteronism type II (PHA II) or Gordon syndrome is characterized by hyperkalemia, hypertension, hyperchloremic metabolic acidosis, low plasma renin activity, and normal kidney function. We report a rare case of a young adult female patient presenting with abdominal pain, diarrhea, and vomiting. She was hypertensive during the presentation. Blood work showed mild anemia, hyperkalemia, hyperchloremia, and metabolic acidosis, with normal renal function and liver function. Plasma renin activity and aldosterone levels were low-normal. These findings were suggestive of PHA II or Gordon syndrome. It is a rare familial disease, with a non-specific presentation and no specific diagnostic criteria, and physicians should suspect it in patients with hyperkalemia in the setting of normal glomerular filtration, along with hypertension (which can be absent), metabolic acidosis, hyperchloremia, low plasma renin activity, and relatively suppressed aldosterone.

## Introduction

Pseudohypoaldosteronism type II (PHA II) or Gordon syndrome is a rare disease with no known prevalence data [1]. It was first reported in the 1960s [2]. Richard Gordan reported the second case as a syndrome of hypertension and hyperkalemia with a normal glomerular filtration rate [3]. It is characterized by hypertension and hyperkalemia but otherwise normal kidney function. Other features described were hyperchloremic metabolic acidosis, low plasma renin activity, and relatively low serum aldosterone levels. It is usually inherited as autosomal dominant, with a variable age of onset [4,5]. Here, we report a rare case of PHA II in an adolescent patient who presented with hypertension and hyperkalemia in the setting of normal renal function.

This article was previously submitted as a meeting abstract and presented as a poster at the ENDO Society in June 2020, and later, the abstract was published in the Journal of the Endocrine Society in 2021.

## Case presentation

A 16-year-old female with no significant past medical history, except for asthma and anemia, presented to the emergency room with severe acute abdominal pain, watery diarrhea, vomiting, and diaphoresis. She was hypertensive at 190/110 mmHg on presentation. A complete blood count showed mild anemia but a normal white count and platelets. The comprehensive metabolic panel showed hyperkalemia (potassium 6.6 mEq/L), hyperchloremia (chloride 115 mEq/L), metabolic acidosis (bicarbonate 16 mEq/L), normal renal function (creatinine 0.5 mg/dL), and normal liver enzymes. Urine electrolyte studies were as follows: sodium 189 mmol/L, potassium 20.8 mmol/L, and chloride 140 mmol/L. Arterial blood gas showed acidosis with a pH of 7.32. Plasma renin activity was low-normal at 0.34 ng/mL/h (normal: 0.25-5.82 ng/mL/h), and aldosterone level was normal at 2 pg/mL (normal: <21 pg/mL) but this was relatively low given the degree of hyperkalemia. Computed tomography of the abdomen and pelvis did not show any abnormalities. The clinical presentation with hypertension and the blood work showing hyperkalemia in the setting of normal renal function, hyperchloremic metabolic acidosis, low plasma renin activity, and relatively low serum aldosterone levels were suggestive of PHA II or Gordon syndrome. The family history was not known as the patient was adopted during early childhood. She is currently being treated with a thiazide diuretic and has well-controlled blood pressure and normal electrolytes.

## Discussion

PHA II or Gordon syndrome is a rare inherited syndrome characterized by hyperkalemia and otherwise normal kidney function [6]. It is frequently associated with hypertension, metabolic acidosis, and hyperchloremia. Plasma renin activity is low or low-normal and aldosterone concentrations are variable but usually low given the degree of hyperkalemia [6]. This contrasts with pseudohypoaldosteronism type I, which is associated with aldosterone resistance [7].

The prevalence of PHA II is unknown. It is frequently inherited as autosomal dominant but can also be inherited as autosomal recessive. Due to a strong family history of similar findings, it is also known as familial hyperkalemic hypertension. It is associated with pathogenic variants of the WNK1, WNK4, CUL3, or KLHL3 genes [4]. The age of onset of PHA II is variable, ranging from the first year of life to adulthood.

The hypothesis for the pathogenesis of PHA II is the abnormal serine/threonine kinase proteins affecting the thiazide-sensitive sodium-chloride cotransporter in the distal nephron [8]. This results in increased sodium and chloride reabsorption, leading to hypertension, hyperchloremia, and volume expansion, resulting in decreased potassium and hydrogen excretion in the distal nephron, leading to hyperkalemia and metabolic acidosis [5,9].

There are no formal diagnostic criteria for PHA II. It should be suspected in individuals, children, or adults with the following findings: hyperkalemia in the absence of impaired renal function (the most consistent finding), hypertension (which can be absent), metabolic acidosis, hyperchloremia, low plasma renin levels, and variable serum aldosterone levels (relatively low in the setting of hyperkalemia) [5]. Hypercalciuria is noted in some patients [10]. Other features reported in this syndrome include short stature, myalgias, periodic paralysis, and dental abnormalities [6]. Usually, a family history of similar findings is present, but the absence of a family history does not preclude the diagnosis [5]. In addition to these clinical features, laboratory findings, and family history, molecular genetic testing can help in the diagnosis by identifying pathogenic variants of the WNK1, WNK4, CUL3, or KLHL3 genes [4].

The management of PHA II includes correction of electrolyte abnormalities, blood pressure control, prevention of secondary complications, and genetic counseling. Electrolyte abnormality correction and blood pressure control are achieved by thiazide diuretics, usually within a week [5]. Sometimes, additional antihypertensives can be used for adequate blood pressure control to reduce the risk of secondary complications like cardiovascular disease, renal disease, or stroke. The patient will require continued surveillance with routine electrolyte and blood pressure measurements, usually every six months to one year. The patient should avoid excessive intake of foods high in salt and potassium [3]. It is recommended to evaluate at-risk relatives with serum potassium levels and blood pressure measurements so that prompt treatment can be initiated. Genetic counseling should be offered to the patients to help them make informed decisions [5]. Table 1 below offers a brief review of PHA II or Gordan syndrome.

**Table 1:** PHA II or Gordon syndrome: a review PHA II: pseudohypoaldosteronism type II

PHA II or Gordon syndrome	
Rare (no known prevalence data), autosomal dominant (mostly)	
Pathogenesis	Mutation in WNK1, WNK4, CUL3, or KLHL3 genes resulting in abnormal serine/threonine kinase proteins affecting the thiazide-sensitive sodium-chloride cotransporter in the distal nephron
Clinical presentation	Non-specific, hypertension (not always)
Laboratory findings	Hyperkalemia, hyperchloremic metabolic acidosis, hypercalciuria (sometimes), low or low-normal plasma renin activity, low serum aldosterone levels (relative to hyperkalemia) with normal kidney function/normal glomerular filtration
Management	Correction of electrolyte abnormalities, salt restriction, thiazide diuretics

## Conclusions

PHA II or Gordon syndrome is a rare familial disease with unknown prevalence. It has a strong genetic preponderance and usually runs in families. It affects Na-Cl cotransporters in the distal nephron and results in increased sodium and chloride reabsorption, leading to hypertension, hyperchloremia, hyperkalemia, and metabolic acidosis in the setting of normal kidney function. The management mainly involves correcting electrolyte abnormalities and treating hypertension, usually with thiazides.

PHA II or Gordon syndrome is a rare disease with a non-specific clinical presentation and no clear-cut diagnostic criteria. Therefore, it should be highly suspected in patients with hypertension and hyperkalemia with normal glomerular filtration.
